# Supplementary material for: Loss of E-cadherin is causal to pathologic changes in chronic lung disease
Source: Commun Biol. 2022 Oct 29;5:1149. doi: 10.1038/s42003-022-04150-w (PMC9617938; doi:10.1038/s42003-022-04150-w)
Supplement: Supplementary file 2 — Supplementary Information-New [file 42003_2022_4150_MOESM2_ESM.pdf]

Supplementary Information

Table S1: Primer details for genotyping the Mice

Gene	Primer (5'-3')	Product details
Ager *(Alveolar Type I cells)*	ATC GCA TTC CTT GCA AAA GT<br>GGA CTC TTG TCC CAG AAG CA	Mutant - ~500 bp<br>Wild type – 200 bp
Cdh1 *(E-cadherin)*	GGG TCT CAC CGT AGT CCT CA<br>GAT CTT TGG GAG AGC AGT CG	Mutant – 310 bp<br>Wild type – 243 bp
Foxj1 *(Ciliated cells)*	P1: GCA GAT GGA GAG AGG TGG AG<br>P2: CTT GGC GTT GAG AAT GGA GA<br>P3: ATT GCA TCG CAT TGT CTG AG	Mutant – 294 bp (P2 & P3)<br>Heterozygote – 294 bp & 472 bp<br>Wild type – 472 bp (P1 & P2)
Rosa *(Cdh1 conditional knock-in)*	CAC TTG CTC TCC CAA AGT CGC TC<br>ATA CTC CGA GGC GGA TCA CAA<br>GAT GGG GAG AGT GAA GCA GAA CG	Mutant – 508 bp<br>Wild-type – 335 bp
Scbg1a1 *(Club cells)*	ACT CAC TAT TGG GGG TGT GG<br>CCA AAA GAC GGC AAT ATG GT	Mutant – 245 bp
Spc *(Alveolar type II cells)*	ATG TCC AAT TTA CTG ACC G<br>CGC GCC TGA AGA TAT AGA AG	~250 bp

Table S2: qPCR primer details to evaluate EMT and fibrosis markers

Gene	Primer	Product details
Human		
CDH1	F: GCC TCC TGA AAA GAG AGT GGA AG<br>R: TGG CAG TGT CTC TCC AAA TCC G	131 bp
GAPDH	F: GTC TCC TCT GAC TTC AAC AGC G<br>R: ACC ACC CTG TTG CTG TAG CCA A	131 bp
Mouse		
Cdh1	F: CAT CAC TGC CAC CCA GAA GAC TG<br>R: ATG CCA GTG AGC TTC CCG TTC AG	153 bp
Cdh2	F: CCT CCA GAG TTT ACT GCC ATG AC<br>R: GTA GGA TCT CCG CCA CTG ATT C	149 bp
Col3a1	F: GAC CAA AAG GTG ATG CTG GAC AG<br>R: CAA GAC CTC GTG CTC CAG TTA G	114 bp
Gapdh	F: CAT CAC TGC CAC CCA GAA GAC TG<br>R: ATG CCA GTG AGC TTC CCG TTC AG	153 bp
Slug/Snai2	F: TCT GTG GCA AGG CTT TCT CCA G<br>R: TGC AGA TGT GCC CTC AGG TTT G	133 bp
Snai1	F: TGT CTG CAC GAC CTG TGG AAA G<br>R: CTT CAC ATC CGA GTG GGT TTG G	163 bp
Twist1	F: GAT TCA GAC CCT CAA ACT GGC G<br>R: AGA CGG AGA AGG CGT AGC TGA G	134 bp

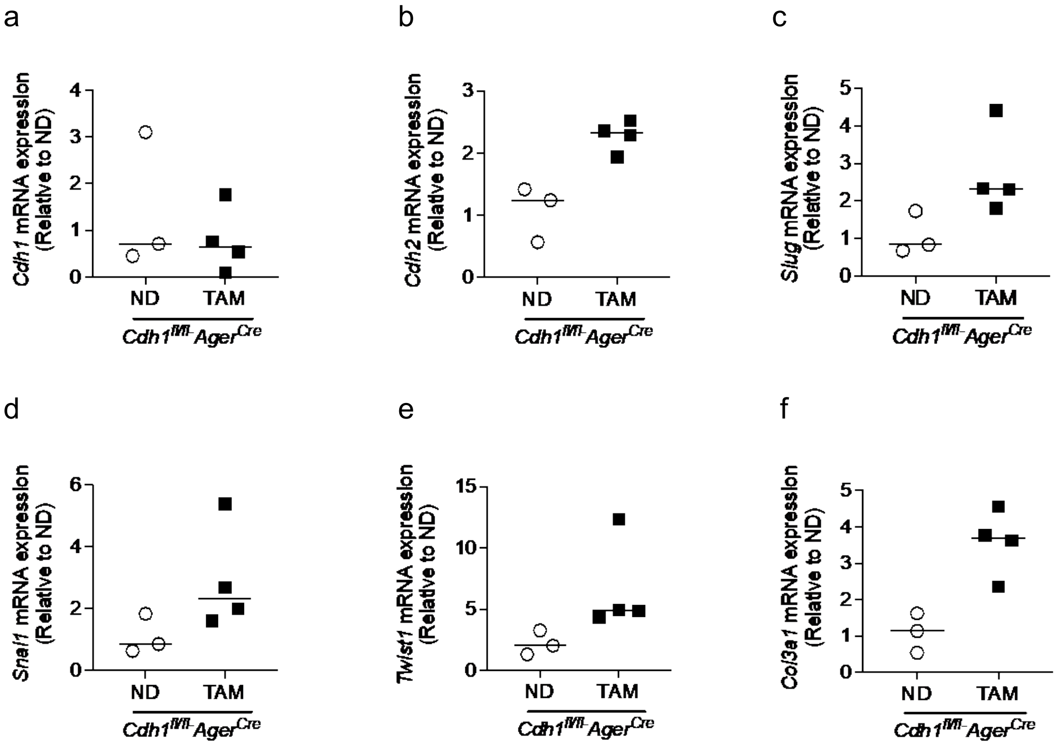

Fig. S1: Increased mRNA expression of mesenchymal and collagen markers in E-cadherin knockdown in AT1 cells of mice. To knock down E-cadherin in the AT1 cells of mice lungs, *Cdh1*$^{fl/fl}$-*Ager*^{Cre} mice were fed a tamoxifen diet (TAM) for 30 days. These were compared to *Cdh1*$^{fl/fl}$-*Ager*^{Cre} mice receiving a normal chow diet (ND). Basal mRNA expression of **(a)** epithelial – *Cdh1* was not altered, whereas mesenchymal – **(b)** *Cdh2*, **(c)** *Slug*, **(d)** *Snai1*, and **(e)** *Twist1* and collagen **(f)** *Col3a1* markers were increased after E-cadherin was knocked down in AT1 cells. Data is expressed as median bars and representative of 3 to 4 mice per group. Mann-Whitney test was performed and $P < 0.05$ was considered statistically significant.

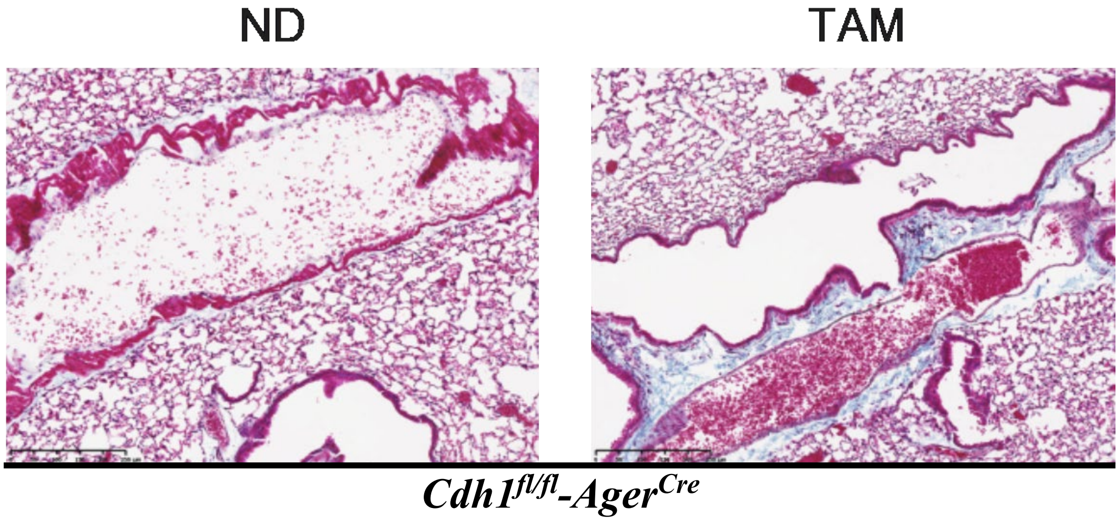

Fig. S2. E-cadherin knockdown in AT1 cells causes fibrosis. To knock down E-cadherin in the AT1 cells of mice lungs, *Cdh1*$^{fl/fl}$-*Ager*^{Cre} mice were fed a tamoxifen diet (TAM) for 30 days. These were compared to *Cdh1*$^{fl/fl}$-*Ager*^{Cre} mice receiving a normal chow diet (ND). The mice lung sections were stained with Masson trichrome. Mice lungs with E-cadherin knockdown in AT1 cells display areas of fibrosis due to excess deposition of collagen bundles (blue coloration) at 10X (scale bar of 250 μm).

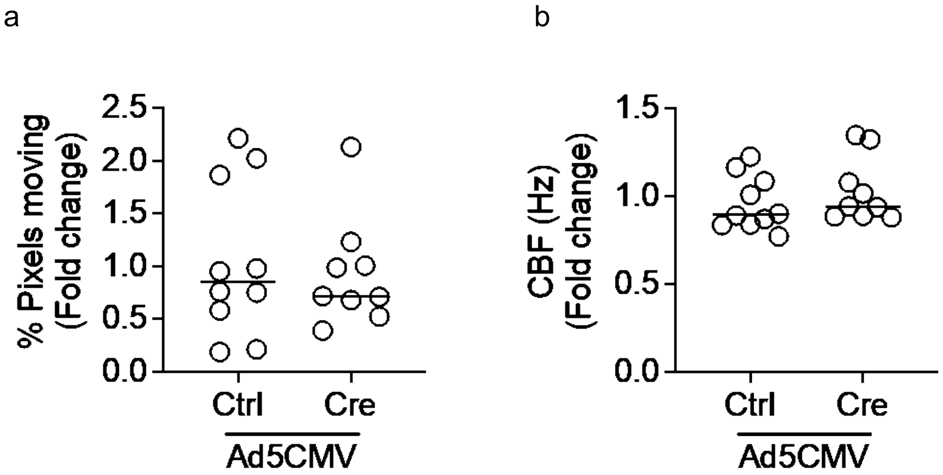

Figure S3: Loss of E-cadherin in mice tracheal epithelial cells (mTECs) does not affect the percentage of the ciliated cells and its ciliary beat frequency (CBF). To knock down E-cadherin in airways, mTECs from *Cdh1*$^{fl/fl}$ mice cultured at the air-liquid interface (ALI) were transfected with Ad5CMVCre-eGFP (Cre) at 2×10^9 pfu and these were compared to Ad-5CMVeGFP (Ctrl). **(a)** Percentage of ciliated cells (% pixels moving) and **(b)** CBF were not altered due to knockdown of E-cadherin. Data is expressed as median bars and representative of cells derived from 12 mice and 9 to 10 inserts. Mann-Whitney test was performed, and $P<0.05$ was statistically significant.

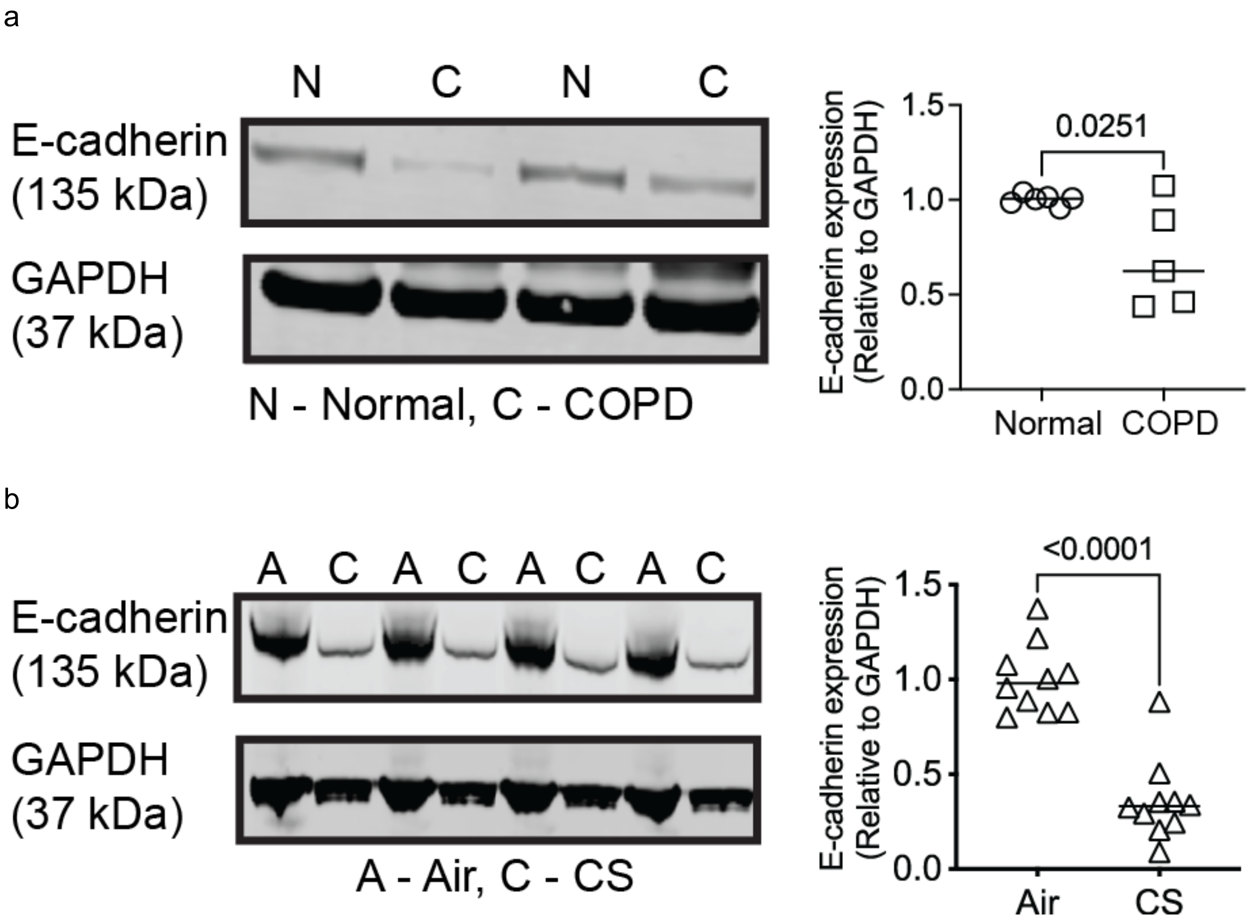

Figure S4. E-cadherin is reduced in COPD and cigarette-smoke (CS) injured epithelium. (a) COPD human bronchial epithelial cells have reduced expression of E-cadherin as compared to age, and sex-matched non-diseased human bronchial epithelial (Normal) cells (representative image – left panel, and quantified blot – right panel). Data is expressed as median bars and representative of 2 to 3 inserts from 2 donors. **(b)** Normal cells exposed to cigarette smoke (CS) demonstrate reduced E-cadherin expression as compared to air control (representative image – left panel, and quantified blot – right panel). Data is expressed as median bars and representative of 3 to 4 inserts from 3 donors. Mann-Whitney test was performed and $P < 0.05$ was considered statistically significant.

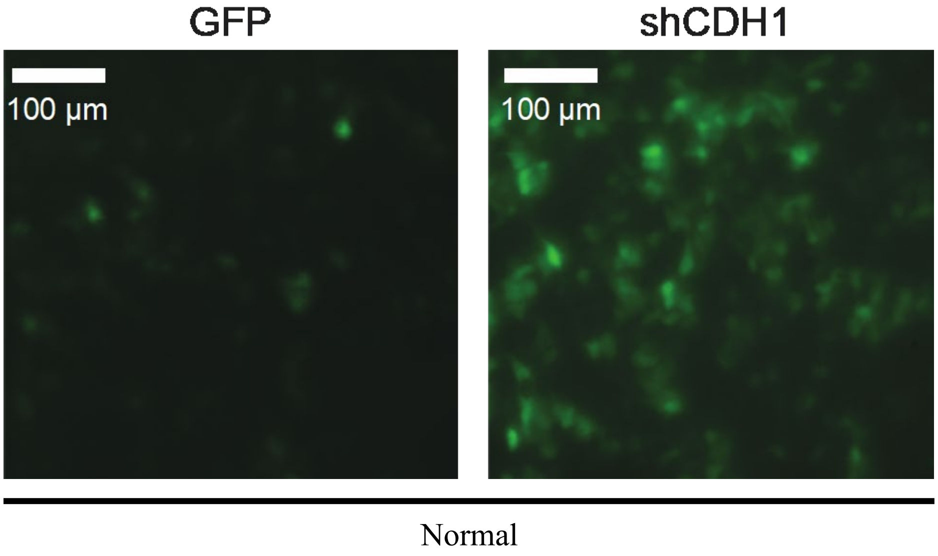

Figure S5: Representative image of Green Fluorescent Protein (GFP) at 20X of the non-diseased airway epithelium (Normal) at 4 to 6 weeks air-liquid interface (ALI) transfected with Adenovirus to knock out E-cadherin. Normal cells were transfected with Ad-GFP-U6-h-CFL1-shRNA (shCDH1) at 1.5×10^9 pfu to knock down E-cadherin and these were compared to control adenovirus (Ad-GFP-U6-shRNA, GFP).

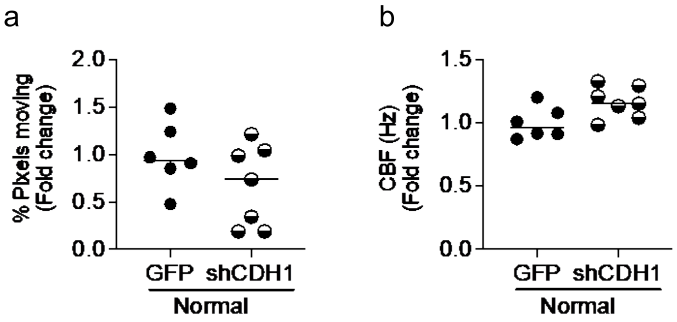

Figure S6: Loss of E-cadherin in non-diseased airway epithelia does not affect the percentage of the ciliated cells and its ciliary beat frequency (CBF). Normal cells were transfected with Ad-GFP-U6-h-CFL1-shRNA (shCDH1) at 1.5×10^9 pfu to knock down E-cadherin and these were compared to control adenovirus (Ad-GFP-U6-shRNA, GFP). **(a)** Percentage of ciliated cells (% pixels moving) and **(b)** CBF were not altered due to knockdown of E-cadherin in Normal. Data is expressed as median bars and representative of 3 donors and 2 to 3 inserts per donor. Mann-Whitney test was performed and $P < 0.05$ was considered statistically significant.

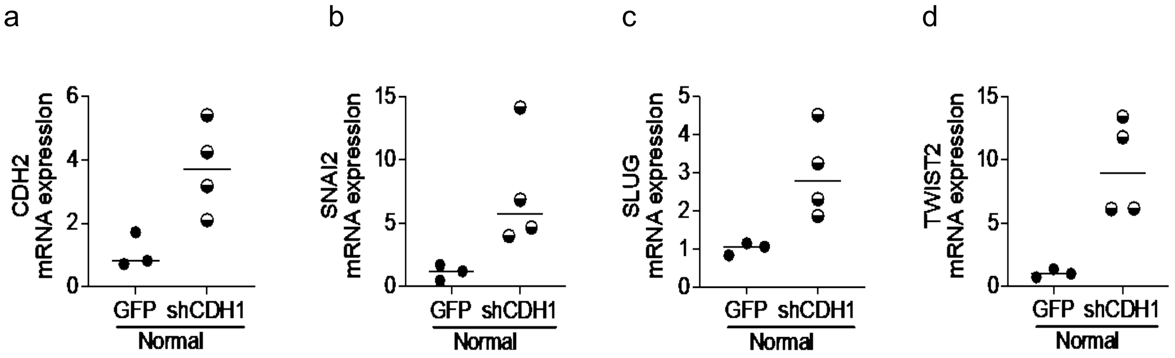

Figure S7: Loss of E-cadherin in non-diseased airway epithelia (Normal) increases mesenchymal markers. Normal cells were transfected with Ad-GFP-U6-h-CFL1-shRNA (shCDH1) at 1.5×10^9 pfu to knock down E-cadherin and these were compared to control adenovirus (Ad-GFP-U6-shRNA, GFP). Normal cells with E-cadherin knockdown showed an increase in mesenchymal markers such as **(a)** *CDH2*, **(b)** *SNAI2*, **(c)** *SLUG*, and **(d)** *TWIST2*. Data is expressed as median bars and representative of 3 donors and 1 insert per donor. Mann-Whitney test was performed and $P < 0.05$ was considered statistically significant.

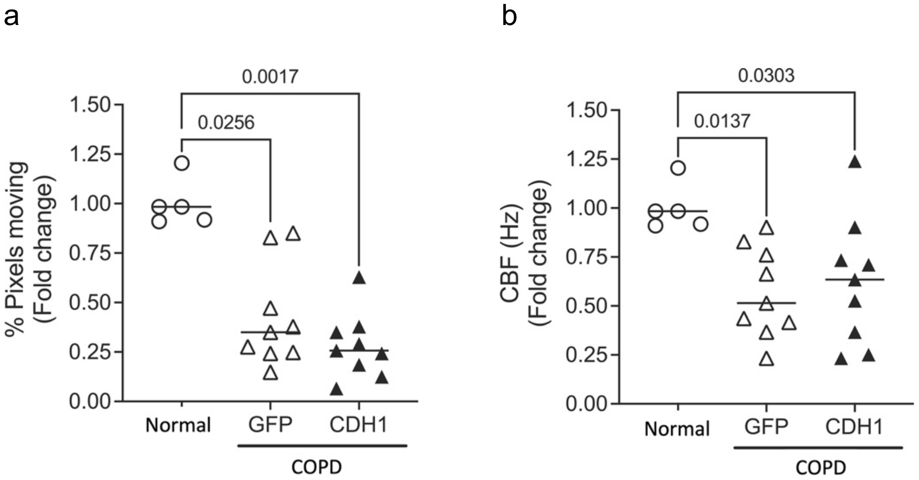

Fig. S8: Overexpression of E-cadherin in COPD epithelia does not affect the percentage of the ciliated cells and its ciliary beat frequency (CBF). COPD at 4 to 6 weeks ALI were transfected Ad-GFP-U6-h-CDH1 (CDH1) to overexpress E-cadherin or Ad-GFP (GFP) as control at 2×10^9 pfu. Transfection of COPD with CDH1 did not alter **(a)** percentage of ciliated cells (% pixels moving) and **(b)** CBF, as compared to COPD, transfected to GFP and age- and gender-matched Normal cells. Data is expressed as median bars and representative of 5 to 9 inserts from 2 donors. Kruskal-Walli's test followed by Dunn's multiple comparison test was performed. $P <$ 0.05 were considered statistically significant.

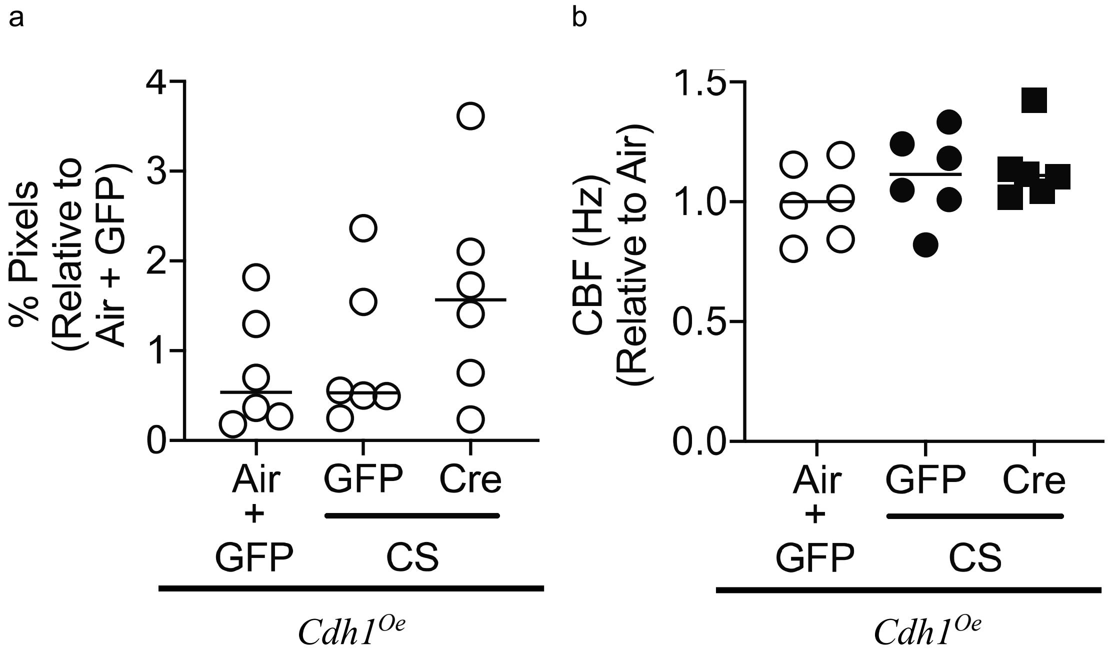

Fig. S9: E-cadherin knock-in mice tracheal epithelial cells (mTECs) does not affect the percentage of the ciliated cells and its ciliary beat frequency (CBF). To induce the over-expression of E-cadherin in Cdh1 knock-in mice (*$Cdh1^{Oe}$*), mTECs were transfected with adeno-Cre (Ad5CMVCre-eGFP) at 2 × 10^9 pfu and were exposed to CS for 10 days. Knock-in of E-cadherin in mTECs did not alter **(a)** percentage of ciliated cells (% pixels moving) and **(b)** CBF. Data is expressed as median bars and representative of 6 inserts from 6 mice. Shapiro-Wilk test followed by Kruskal-Wallis multiple comparison tests was performed, and $P < 0.05$ were considered statistically significant.

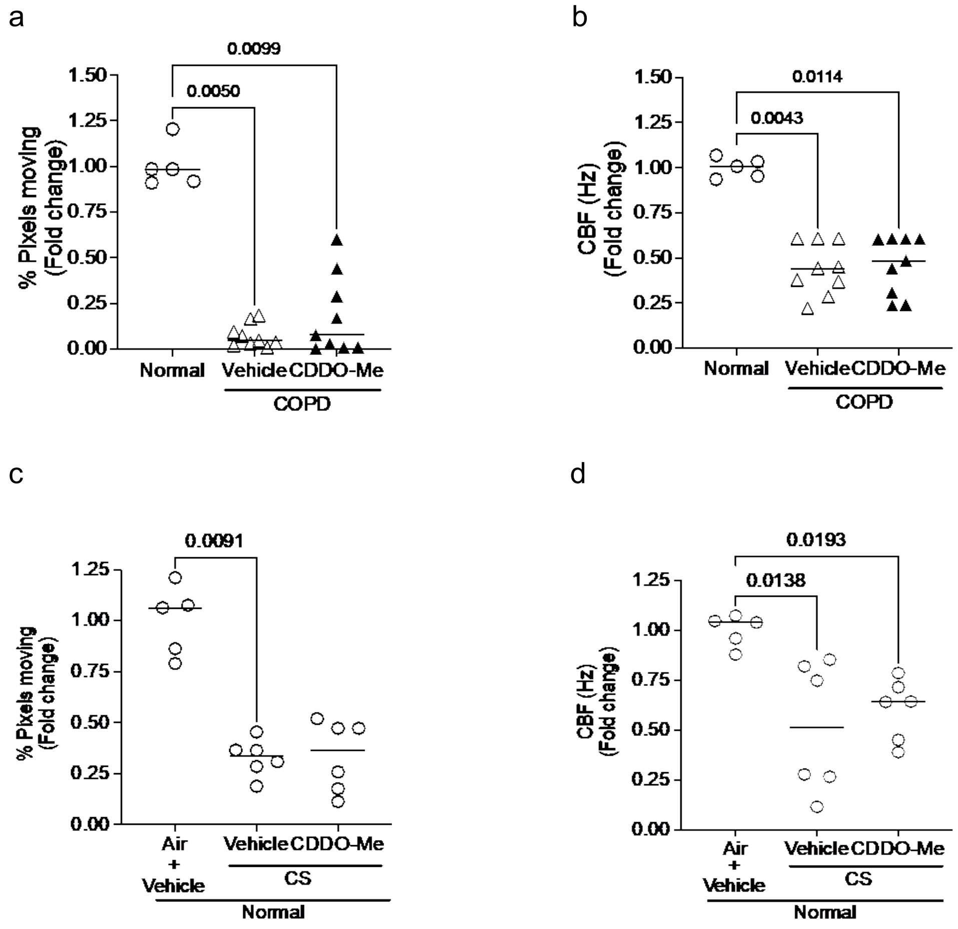

Fig. S10: Nrf2-activator does not protect loss of cilia or decreased ciliary beat frequency in COPD and cigarette-smoke exposed epithelia. The COPD or the Normal epithelial cells exposed to cigarette smoke (CS) were treated with 100 nM of CDDO-Me (a Nrf2 pathway activator) for 5 days and 10 days respectively. COPD treated with CDDO-Me did not restore **(a)** percentage of ciliated cells (% pixels moving) and **(b)** ciliary beat frequency as compared to non-diseased epithelial cells. Similarly, CS exposed Normal cells treated with CDDO-Me did not restore CS induces **(c)** loss of ciliated cells and **(d)** CBF as compared to air exposed Normal cells. Data is representative of 5 to 9 inserts from 2 donors. Shapiro-Wilk test followed by Kruskal-Wallis multiple comparison tests was performed. $P < 0.05$ were considered statistically significant.

Uncropped gel blots for Fig. 6B

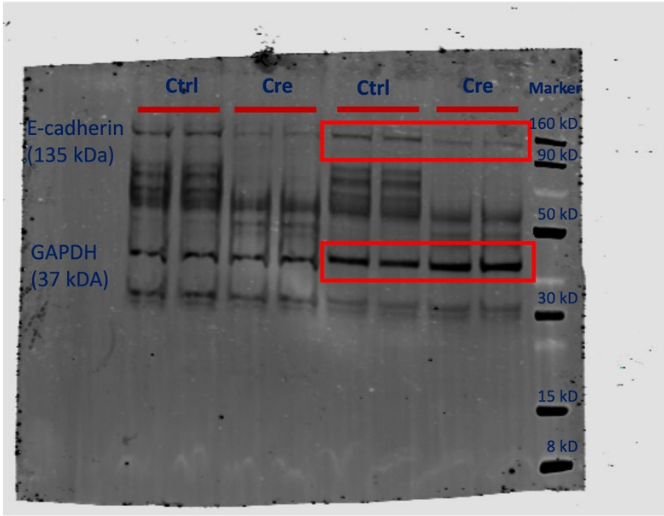

Uncropped gel blots for Fig. 6F

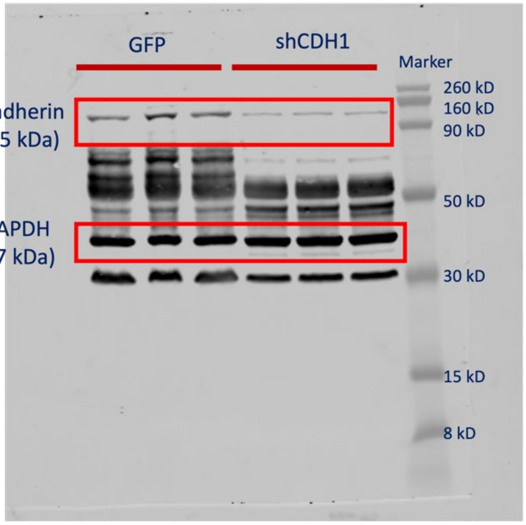

Uncropped gel blots for Fig. S4A

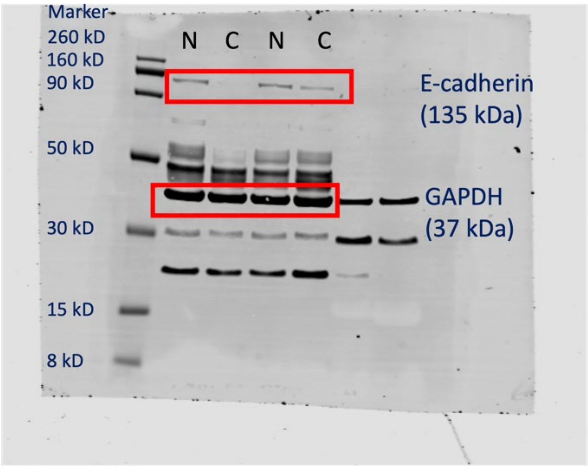

Uncropped gel blots for Fig. S4B

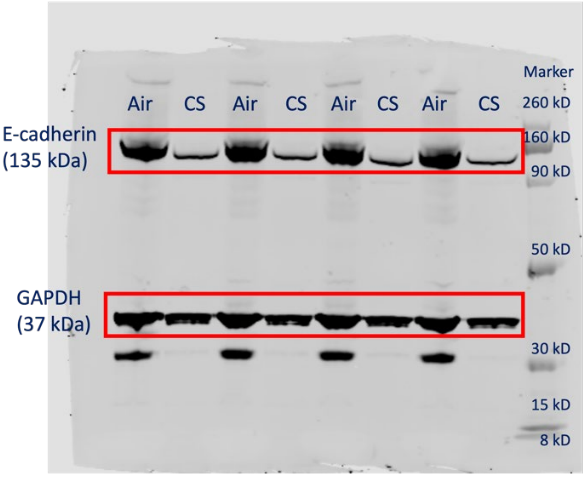

Fig. S11: Original and uncropped gel blots of manuscript. Red border indicates the lanes used in manuscript.
