# Supplementary material for: Loss of E-cadherin is causal to pathologic changes in chronic lung disease
Source: Commun Biol. 2022 Oct 29;5:1149. doi: 10.1038/s42003-022-04150-w (PMC9617938; doi:10.1038/s42003-022-04150-w)
Supplement: Supplementary file 3 — Description of Additional Supplementary Files [file 42003_2022_4150_MOESM3_ESM.docx]

**Description of Additional Supplementary Files**

**File name:** Supplementary Data 1
**Description:** Supplementary Data 1 consists of source data behind the graphs in the article file.

**File name:** Supplementary Data 2
**Description:** Supplementary Data 2 consists of source data behind the graphs in the supplementary information.
