# Supplementary material for: Loss of E-cadherin is causal to pathologic changes in chronic lung disease
Source: Commun Biol. 2022 Oct 29;5:1149. doi: 10.1038/s42003-022-04150-w (PMC9617938; doi:10.1038/s42003-022-04150-w)
Supplement: Supplementary file 4 — Reporting Summary [file 42003_2022_4150_MOESM4_ESM.pdf]

nature portfolio

Corresponding author(s): Venkataramana Sidhaye

Last updated by author(s): Jan 24, 2022

Reporting Summary

Nature Portfolio wishes to improve the reproducibility of the work that we publish. This form provides structure for consistency and transparency in reporting. For further information on Nature Portfolio policies, see our Editorial Policies and the Editorial Policy Checklist.

Statistics

For all statistical analyses, confirm that the following items are present in the figure legend, table legend, main text, or Methods section.

n/a	Confirmed	
☐	☒	The exact sample size (*n*) for each experimental group/condition, given as a discrete number and unit of measurement
☐	☒	A statement on whether measurements were taken from distinct samples or whether the same sample was measured repeatedly
☐	☒	The statistical test(s) used AND whether they are one- or two-sided<br>*Only common tests should be described solely by name; describe more complex techniques in the Methods section.*
☐	☒	A description of all covariates tested
☐	☒	A description of any assumptions or corrections, such as tests of normality and adjustment for multiple comparisons
☐	☒	A full description of the statistical parameters including central tendency (e.g. means) or other basic estimates (e.g. regression coefficient) AND variation (e.g. standard deviation) or associated estimates of uncertainty (e.g. confidence intervals)
☐	☒	For null hypothesis testing, the test statistic (e.g. *F*, *t*, *r*) with confidence intervals, effect sizes, degrees of freedom and *P* value noted<br>*Give P values as exact values whenever suitable.*
☒	☐	For Bayesian analysis, information on the choice of priors and Markov chain Monte Carlo settings
☒	☐	For hierarchical and complex designs, identification of the appropriate level for tests and full reporting of outcomes
☒	☐	Estimates of effect sizes (e.g. Cohen's *d*, Pearson's *r*), indicating how they were calculated

Our web collection on statistics for biologists contains articles on many of the points above.

Software and code

Policy information about availability of computer code

Data collection	Not applicable
Data analysis	Microsoft excel for Mac (Version 16.57), MATLAB R2020a Update 7 (9.8.0.17217073), GraphPad Prism 9 (Version 9.3.1 (350)), STEPanizer1-8.jar, Image Studio Lite (Version 5.2.5)

For manuscripts utilizing custom algorithms or software that are central to the research but not yet described in published literature, software must be made available to editors and reviewers. We strongly encourage code deposition in a community repository (e.g. GitHub). See the Nature Portfolio guidelines for submitting code & software for further information.

Data

Policy information about availability of data

All manuscripts must include a data availability statement. This statement should provide the following information, where applicable:

- Accession codes, unique identifiers, or web links for publicly available datasets
- A description of any restrictions on data availability
- For clinical datasets or third party data, please ensure that the statement adheres to our policy

This published article and its supplementary information files include all data generated or analyzed during this study. Raw data is available upon submission of request to vsidhay1@jhmi.edu or bghosh5@jh.edu.

Field-specific reporting

Please select the one below that is the best fit for your research. If you are not sure, read the appropriate sections before making your selection.

☒ Life sciences ☐ Behavioural & social sciences ☐ Ecological, evolutionary & environmental sciences

For a reference copy of the document with all sections, see nature.com/documents/nr-reporting-summary-flat.pdf

Life sciences study design

All studies must disclose on these points even when the disclosure is negative.

Sample size	Sample size are described in the the figure legends for each figure in the manuscript. The sample sizes was chosen based on previous experience for each experiment to yield high power to detect specific effects. For both in vivo and in vitro mice model experiments n ≥ 5.
Data exclusions	No data or samples was excluded from the study.
Replication	The experiments in this study were repeated and experimental findings were reproducible.
Randomization	Animals were randomly assigned.
Blinding	The investigators were blinded during the data collection for mice physiological measurements , and mean linear intercept. The data collection for epithelial resistance and permeability, ciliary function and cellular velocity were not blinded in this study. Also, the data analysis was not perform by blinding.

Reporting for specific materials, systems and methods

We require information from authors about some types of materials, experimental systems and methods used in many studies. Here, indicate whether each material, system or method listed is relevant to your study. If you are not sure if a list item applies to your research, read the appropriate section before selecting a response.

Materials & experimental systems

n/a	Involved in the study	
☐	☒	Antibodies
☒	☐	Eukaryotic cell lines
☒	☐	Palaeontology and archaeology
☐	☒	Animals and other organisms
☒	☐	Human research participants
☒	☐	Clinical data
☒	☐	Dual use research of concern

Methods

n/a	Involved in the study	
☒	☐	ChIP-seq
☒	☐	Flow cytometry
☒	☐	MRI-based neuroimaging

Antibodies

Antibodies used

Antibodies from Abcam: (1) Ms mAb to BrdU - Anti-BrdU antibody [IIB5] (ab8152) (Lot no.: GR3404329-1); (2) Recombinant Anti-Prosurfactant Protein C antibody [EPR19839] (ab211326) (Lot no.: GR3247122-5).

Antibodies from Cell Signaling Technology (CST): (3) E-cadherin (24E10) Rabbit mAb #3195 (Lot no.: 15); (4) E-cadherin (4A2) Mouse mAb #14472; (5) β-Tubulin (D2N5G) Rabbit mAb #15115; (6) GAPDH (14C10) Rabbit mAb #2118 (Lot:8).

Antibodies from SantaCruz: (7) Anti-Cytokeratin 14 Antibody (LL001): sc-53253; Recombinant Anti-Mucin 5AC antibody [45MI]:ab3649.

Antibodies from ThermoFisher Scientific: (9) Goat anti-Rabbit IgG (H+L) Cross-Adsorbed Secondary Antibody, Alexa Fluor 647 (A21244) (Lot no.: 792514); (10) Goat anti-Rabbit IgG (H+L) Highly Cross-Adsorbed Secondary Antibody, Alexa Fluor 488 (A11034) (Lot No.: 2156517); (11) Goat anti-Rabbit IgG (H+L) Cross-Adsorbed Secondary Antibody, Alexa Fluor 555 (A21428) (Lot No.: 1937183); (12) Goat anti-Mouse IgG (H+L) Highly Cross-Adsorbed Secondary Antibody, Alexa Fluor 647 (A21236) (Lot No.: 2170302); (13) Goat anti-Mouse IgG (H+L) Cross-Adsorbed Secondary Antibody, Alexa Fluor 488 (A11001) (Lot No.: 1907294)

Antibodies from LI-COR: IRDye® 800CW Goat anti-Rabbit IgG Secondary Antibody (926-32211)

Validation

All antibodies were verified by the supplier and each lot has been quality tested by the supplier.
1. Ms mAb to BrdU - Anti-BrdU antibody [IIB5] (ab8152). Validation details available at: https://www.abcam.com/brdu-antibody-iib5-ab8152.html
2. Recombinant Anti-Prosurfactant Protein C antibody [EPR19839] (ab211326). Validation details available at: https://www.abcam.com/prosurfactant-protein-c-antibody-epr19839-ab211326.html

3. E-cadherin (24E10) Rabbit mAb #3195. Validation details available at: https://en.cellsignal.jp/products/primary-antibodies/e-cadherin-24e10-rabbit-mab/3195?site-search-type=Products&N=4294956287&Ntt=e-cadherin&fromPage=plp
4. E-cadherin (4A2) Mouse mAb #14472. Validation details available at: https://www.cellsignal.com/products/primary-antibodies/e-cadherin-4a2-mouse-mab/14472
5. β-Tubulin (D2N5G) Rabbit mAb #15115. Validation details available at: https://www.cellsignal.com/products/primary-antibodies/b-tubulin-d2n5g-rabbit-mab/15115
6. GAPDH (14C10) Rabbit mAb #2118. Validation details available at: https://en.cellsignal.jp/products/primary-antibodies/gapdh-14c10-rabbit-mab/2118?site-searchtype=Products&N=4294956287&Ntt=gapdh&fromPage=plp
7. Anti-Cytokeratin 14 Antibody (LL001): sc-53253. Validation details available at: https://www.scbt.com/p/cytokeratin-14-antibody-ll001
8. Recombinant Anti-Mucin 5AC antibody [45MI]:ab3649. Validation details available at: https://www.abcam.com/mucin-5ac-antibody-45m1-ab3649.html
9. Goat anti-Rabbit IgG (H+L) Cross-Adsorbed Secondary Antibody, Alexa Fluor 647 (A21244). Validation details available at: https://www.thermofisher.com/antibody/product/Goat-anti-Rabbit-IgG-H-L-Cross-Adsorbed-Secondary-Antibody-Polyclonal/A-21244
10. Goat anti-Rabbit IgG (H+L) Highly Cross-Adsorbed Secondary Antibody, Alexa Fluor 488 (A11034). Validation details available at: https://www.thermofisher.com/antibody/product/Goat-anti-Rabbit-IgG-H-L-Highly-Cross-Adsorbed-Secondary-Antibody-Polyclonal/A-11034
11. Goat anti-Rabbit IgG (H+L) Cross-Adsorbed Secondary Antibody, Alexa Fluor 555 (A21428). Validation details available at: https://www.thermofisher.com/antibody/product/Goat-anti-Rabbit-IgG-H-L-Cross-Adsorbed-Secondary-Antibody-Polyclonal/A-21428
12. Goat anti-Mouse IgG (H+L) Highly Cross-Adsorbed Secondary Antibody, Alexa Fluor 647 (A21236). Validation details available at: https://www.thermofisher.com/antibody/product/Goat-anti-Mouse-IgG-H-L-Highly-Cross-Adsorbed-Secondary-Antibody-Polyclonal/A-21236
13. Goat anti-Mouse IgG (H+L) Cross-Adsorbed Secondary Antibody, Alexa Fluor 488 (A11001). Validation details available at: https://www.thermofisher.com/antibody/product/Goat-anti-Mouse-IgG-H-L-Cross-Adsorbed-Secondary-Antibody-Polyclonal/A-11001

Animals and other organisms

Policy information about studies involving animals; ARRIVE guidelines recommended for reporting animal research

Laboratory animals

The study consisted of Cdh1fl/fl (BALB/c), Ager-CreERT2 (C57BL/6), Foxj1-CreERT2 (BALB/c), Scbg1-CreERTM (C57BL/6NJ), and Sftpc-CreERT2 (C57BL/6) mice. We have also created a mouse Cdh1 conditional knock-in (gene for E-cadherin) at the locus of ROSA26 in C57BL/6 mice by CRISPR/Cas-mediated genome engineering. The mice were at least 5 weeks of age prior performing any experiments on them.

Wild animals

Not applicable

Field-collected samples

Not applicable

Ethics oversight

The study was approved by the Institutional Animal Care and Use Committee (IACUC) of the Johns Hopkins University Animal Use and Care Committee and complied with the Guidelines for Care and Use of Laboratory Animals issued by the USA National Institute of Health (Protocol Number: MO19M405).

Note that full information on the approval of the study protocol must also be provided in the manuscript.
